# Update on Treatment of Infantile Hemangiomas: What’s New in the Last Five Years?

**DOI:** 10.3389/fphar.2022.879602

**Published:** 2022-05-26

**Authors:** Laura Macca, Domenica Altavilla, Luca Di Bartolomeo, Natasha Irrera, Francesco Borgia, Federica Li Pomi, Federico Vaccaro, Violetta Squadrito, Francesco Squadrito, Mario Vaccaro

**Affiliations:** ^1^ Department of Clinical and Experimental Medicine, Dermatology, University of Messina, Messina, Italy; ^2^ Department of Clinical and Experimental Medicine, Pharmacology, University of Messina, Messina, Italy; ^3^ Department of Dermatology, University of Modena and Reggio Emilia, Modena, Italy; ^4^ Department of Human Pathology in Adult and Developmental Age “Gaetano Barresi, Pediatry”, University of Messina, Messina, Italy

**Keywords:** infantile hemangioma, steroids, propranolol, timolol, carteolol, itraconazole, pulsed dye laser, segmental hemangioma

## Abstract

Among benign vascular tumors of infancy, hemangiomas are the commonest, affecting approximately 5–10% of one-year-old children. They are derived from a benign proliferation of vascular endothelial cells (VECs) in the mesoderm and may arise anywhere on the body around 1–2 weeks after birth. Infantile hemangiomas (IHs) are characterized by an early proliferative phase in the first year followed by a spontaneous progressive regression within the following 5 years or longer. IH prevalence is estimated to be 5%–10% in one-year-old children and commonly affects female, Caucasian and low-birth weight infants. Although most of them spontaneously regress, approximately 10% requires treatment to prevent complications due to the site of occurrence such as bleeding, ulceration, cosmetically disfigurement, functional impairment, or life-threatening complications. For over 30 years, steroids have represented the first-line treatment for IHs, but recently topical or systemic β-blockers are increasingly being used and recognized as effective and safe. A search for “Cutaneous infantile hemangioma” [All Fields] AND “Treatment” [All Fields] was performed by using PubMed and EMBASE databases. Treatment of IHs with labeled drugs, such as oral propranolol, but also with off-label drugs, such as topical β-blockers, including topical timolol and carteolol, steroids, itraconazole or sirolimus, with a focus on formulations types and adverse events were described in our review. We also discussed the benefits of pulsed dye laser and the treatment of IHs with involvement of central nervous system, namely the PHACE and LUMBAR syndrome.

## Introduction

Infantile hemangiomas (IHs) are the commonest vascular tumors of the children (Munden et al., 2014; Püttgen, 2014). The lesions arise from the benign proliferation of vascular endothelial cells (VECs) in the mesoderm occurring anywhere on the body, more frequently on the head or face 1–2 weeks after birth (Hoornweg et al., 2012). The course of hemangiomas is characterized by a proliferative phase followed by a plateau and a regression phase. During the first year (proliferative phase), IH grow rapidly and may also ulcerate, bleed, or become infected. This phase is followed by gradual spontaneous involution (regression phase) over the next 1–5 years or longer (Chang et al., 2008; Yanes et al., 2016).

IHs can be classified by general appearance: 1) superficial, located in the upper dermis 2) deep, extending to subcutaneous fascia, 3) mixed (Hoornweg et al., 2012) and the diagnosis is predominantly clinical. The estimated incidence of IHs is 1.1–2.6% in newborns, while IH prevalence is estimated to be 5%–10% in one-year-old children and commonly affects female, Caucasian and low-birth weight infants (Püttgen, 2014; Wang et al., 2017; Price et al., 2018). The use of drugs in first-degree relatives increase IHs risk (Li J. et al., 2011). Other predisposing factors associated with IHs are represented by old maternal age, placenta previa and pre-eclampsia (Bauland et al., 2010).

## Materials and Methods

PubMed (https://ncbi.nlm.nih.gov/PubMed) and EMBASE databases were checked by using the string “Cutaneous infantile hemangioma” [All Fields] AND “Treatment” [All Fields]. Only papers written in English language, concerning humans and with 5 years’ time limits were included. The references retrieved were critically examined to select those pertinent, thus reporting the type of the selected articles (review, retrospective cohort study, clinical trial, case series, research studies, case reports). The reference lists of these papers were also examined to find other relevant articles, which were eventually included if appropriate.

## Results

As of 21 October 2021, we found 23 articles of interest. Among these, seven reviews, four retrospective cohort studies, five clinical trials, 2 case series, two research studies and 3 case reports were selected. In Table 1 are summarized the following features of each article: first author, year of publication, type of study, study population and strength level.

**TABLE 1:** Papers concerning treatment of cutaneous infantile hemangioma (CIH).

Publication (authors and year)	Type of study	Study population and treatment outcomes
de Castro et al. (2017)	Retrospective cohort study	18 patients (9 female) with vascular lip anomalies underwent a single or double pentagonal-shaped wedgeresection of the involved upper or lower lip
Hutchins et al. (2017)	Case report	a 3-year-old girl presenting diffuse cutaneous, hepatic and pulmonary IHs with progressive pulmonary hypertension treated with oral sirolimus
Kim et al. (2017)	Clinical trial	34 patients (15 boys, 19 girls) randomized to receive either propranolol or steroid treatment (17 in each treatment group). The treatment response rate in the propranolol group was 95.65%, and that of the steroid group was 91.94%. Propranolol was considered noninferior. There was no difference between the groups in safety outcomes
Mashiah et al. (2017)	Retrospective cohort study	63 patients with a total of 75 IHs treated with topical propranolol 4%. Of the total number of IHs, 43 (57.3%) showed a good response to treatment, 19 (25.3%) a partial response, and 13 (17.33%) poor or no response
Yu et al. (2017)	Case report	A 1-month-old female infant with LUMBAR syndrome treated with oral propranolol (2 mg/day)
Wang et al. (2017)	Clinical trial	40 infants treated with 2% propranolol cream followed up for 12 months after 3-month treatment. Poor response was observed in 2 patients, moderate response in 15 patients, good response in 17 patients, and excellent response in 6 patients
Ying et al. (2017)	Clinical trial	21 patients with superficial IH. Each lesion was divided into two regions; one part was treated with 0.5% topical timolol cream four times daily, and the other part was treated monthly with PDL. Both treatments were continued for 2–6 months. Both treatments resulted in significant clinical improvements after 3.39 sessions in the 2-month follow up
Le Sage et al. (2018)	Case series	6 patients of which 1 with an infantile hemangioma on the right forehead without intracranial extension treated with topical sirolimus 0.1% without improvement
Xu et al. (2018)	Clinical trial	Thirty-five children achieved the treatment of Intralesional Compound Betamethasone, 134 children achieved the treatment of oral propranolol, and 16 children achieved the treatment of topical carteolol. In the follow-up, the treatment could be repeated or switched to oral propranolol if the tumor tended to grow again. At the end of follow- up, 89% of the patients’ tumors shrinked or involuted completely, 5 patients switched to oral propranolol
Igarashi et al. (2018)	Case report	A case of an extremely low-birth-weight infant with massive cutaneous IH complicated with hypothyroidism, which had improvement of hypothyroidism and regression of cutaneous hemangioma after propranolol therapy
Xu et al. (2020)	Retrospective cohort study	Eighty-one lip infantile hemangiomas patients treated with systemic propranolol (2 mg/kg/die). Lesions showed the same outcomes and prognosis involving the upper lips as the lower lips. Lesions involving the vermillion border had longer treatment lengths, poorer outcomes, and prognosis than lesions confined to one side of the vermilion
Chen et al. (2019)	Research study	The inhibitory effects of itraconazole on IH and its underlying molecular mechanisms were explored usingthe endothelial cells of mouse hemangioendothelioma (EOMA) cell line and infantile primary hemangioma endothelialcell (HemEC)
Tani et al. (2020)	Case series	A3-month-old boy with cheek’s hemangioma (mixed type), a 3-month-old boy with forehead and abdomen’s hemangioma (mixed type), a 6-month-old girl with head’s hemangioma (superficial type), a 4-month-old girl with buttock’s hemangioma (superficial type), and a 4-month-old boy with forehead’s hemangioma (deep type). All patients treated with oral propranolol (3 mg/kg/die)
Diociaiuti et al. (2020)	Retrospective cohort study	Seven patients with intracranial or intraspinal infantile hemangiomas were selected and treated with oral propranolol, without side-effects
Zeng et al. (2020)	Research study	PRH-CNPs were tested on abdominal skin from Sprague Dawley (SD) rats to evaluate skin permeation. PRH-CNPs cytotoxicity on endothelial cells of mouse hemangioendothelioma (EOMA) was also assessed
Cheng et al. (2020)	Clinical trial	Forty-two children received 12 months of 0.5% TTM solution (24 assigned to the TTM group and 17 to the no-TTM group) The TTM group had fewer complications than the no-TTM group (4.2% versus 29%). Mean IH volume percentage reduction was significantly more for the and no-TTM group at 3, 6 and 12 months

## Treatment OF IHs

This is the case of a particular rare, self-limited disease featured by multiple cutaneous IHs without markable visceral lesions: benign neonatal hemangiomatosis (BNH). BNH lesions spontaneously subside within 4 months of onset or within the second year of life. In an infant with multiple (>5) cutaneous hemangiomas, it is important to differentiate BNH from diffuse neonatal hemangiomatosis (DNH), which is marked by multiple cutaneous and visceral hemangiomas with mortality ranging from 60% to 81% (Korekawa et al., 2020).

Given that most IHs spontaneously regress, they do not require any treatment, so periodic follow-up is sufficient (Schwartz et al., 2009).

Generally, complications are mild but some IHs can dramatically grow and leave residual cutaneous modifications after the involution phase, including telangiectasia, atrophy, scarring and skin laxity (Bauland et al., 2011; Léauté-Labrèze et al., 2017). Approximately 10% requires treatment to prevent distressing complications due to the site of occurrence such as bleeding, ulceration, visual impairment, eating disorder, airway obstruction, lifelong disfigurement, congestive heart failure, or bowel obstruction (Frieden et al., 2005; Hemangioma Investigator Group et al., 2007; Cheng and Friedlander, 2016; Novoa et al., 2019). In the next paragraphs, we’ll report the last 5-years experiences with propranolol, the first line therapy for IHs treatment, and, in particular, with off-label drugs, such as topical β-blockers, including topical timolol and carteolol, steroids, itraconazole or sirolimus.

## Steroids

For over 30 years, steroids have been used as the first-line therapy for IHs (Zarem and Edgerton, 1967; Folkman, 1984) thanks to their possible antiangiogenic effect. Corticosteroids can be orally, intravenously, intramuscularly, and topically administered. However, their systemic use may lead to various complications including Cushing-like manifestations, adrenal suppression, gastroesophageal reflux and growth disorders, even though such complications are linked with long-term and high dose therapy (George et al., 2004). Topical administration is advantageous, safe with fast response, and is extensively used for small tumors (Yuan et al., 2015). A significant lesion reduction (85.7% response rate) was observed in the study of Xu et al. (2018): 35 out of 185 IHs patients (mean age 3.9 months), presenting small-size hemangiomas which were raised and <3 cm X 3 cm, were treated with intralesional administration of betamethasone (one or two injection of Diprospan 1 ml/ampoule).

## Oral Propranolol

### Oral Propranolol Versus Steroids

Some positive effects of propranolol use for the treatment of IH have been reported. Differently from Zimmermann et al. (2010), the clinical trial lead by Kim et al. (2017) showed non-inferiority of the therapeutic effects of propranolol compared with steroids, with a response rate of 95.65% in the propranolol-treated group and of 91.94% in the steroids-treated one; therefore, no statistically significant difference between the two groups (3.71%) was observed, thus demonstrating that propranolol shows safe outcomes and effectiveness if compared with steroids (Kim et al., 2017).

### Propranolol: Mechanism of Action

The therapeutic effect of oral propranolol administration in IHs was incidentally discovered in 2008 (Léauté-Labrèze et al., 2008). Propranolol is a nonselective beta-blocker that acts on both β-1 and β-2 adrenergic receptors. Several studies demonstrated that the natural course of IHs can be shortened by propranolol-based therapy, which also suppress lesion’s proliferation (Chen et al., 2013; Tan et al., 2015). As its mechanism of action has not been fully understood, it has been suggested that propranolol suppresses both vascular endothelial growth factor (VEGF) (Tang et al., 2015) and vascular endothelial growth factor receptor-2 (VEGFR-2) expression (Lamy et al., 2010). Tani et al. (2020) measured the serum cytokine concentrations of five patients with IH before and during the treatment with propranolol. The authors observed a significant reduction of the platelet-derived growth factor-BB (PDGF-BB) during treatment. This report suggested that PDGF-BB may be involved in propranolol effects and might be considered as a potential marker of the therapeutic effect.

### Propranolol as First Line Treatment

Oral propranolol actually represents the first line approach for the pharmacological treatment of IHs (Hoeger et al., 2015). Oral beta-blocker are typically indicated in case of functional impairment (e.g., periocular IH causing amblyopia, nasal IH causing nose deformity, lip IH leading to feeding difficulties, and auricular IH causing deafness) and to avoid life-threatening complications due to lesion’s locations (e.g., respiratory distress caused by lung IH, airways obstruction caused by subglottic IH, hepatic dysfunction and heart failure subsequent to large cutaneous IH) (Wedgeworth et al., 2016).

In the study conducted by Xu et al. (2018), 134 out of 185 patients received treatment with oral propranolol (1.5 mg/kg/day) and the response rate was 91.7%. The effective dose of oral propranolol is between 1 and 3 mg/kg/day (Léauté-Labrèze et al., 2015).

Oral propranolol may be administered also in cases of IHs and comorbidities, such as hypothyroidism because of the hypotensive effects. Hypothyroidism has been also reported in association with hepatic IHs (Igarashi et al., 2018). Generally, the cells of hepatic IHs lead to thyroid hormone inactivating enzyme type-3 iodothyronine deiodinase (D3) overexpression, causing rapid degradation of thyroid hormones with consequent hypothyroidism (Simsek et al., 2018). Igarashi et al. (2018) reported a case of an extremely low-birth-weight infant with extensive cutaneous IH associated with hypothyroidism. The Authors observed an improvement of hypothyroidism and regression of cutaneous hemangioma following propranolol therapy, thus providing evidence for its effectiveness.

### Adverse Events

The potential propranolol-related AEs generally comprise hypoglycemia, bradycardia, hypotension, bronchospasm, and electrolyte disturbance, even if the overall risk of AEs outbreaks is relatively low, in particular when used at low doses (Hoeger et al., 2015; Tan et al., 2015).

## Topical Propranolol

### Topical Versus Oral Propranolol

Topical propranolol seems to be less effective but safer than oral administration propranolol and particularly helpful in patients that present small superficial hemangiomas, where the aesthetic or asymptomatic impact did not require oral propranolol treatment (Baselga et al., 2016; Zaher et al., 2013). A systematic review identified 12 studies published between 2012 and 2017 and reported that the administration of topical propranolol was the first-line therapy for IHs in over 600 patients, which did not show any systemic side effect. Propranolol preparations comprehend creams, unguents and gels prepared by galenic formulations with a concentration of propranolol ranging from 0.5% to 5%; treatment duration ranged from 2 weeks to 16.5 months. Overall, the initiation of topical propranolol led to lesion’s improvement in 90% of cases, reducing lesion’s size of at least 50% in 59% of IHs (Price et al., 2018).

### Topical Propranolol Formulations

Casiraghi et al. (2016) showed that hydrophilic preparations, such as cream or gel, guarantees higher levels of propranolol permeation than hydrophobic ointments. Mashiah et al. (2017) Fare clic o toccare qui per immettere il testo. observed that 43 out of 65 patients (57.3%), with a total of 75 IHs, had a good response to the treatment with propranolol 4% gel. They observed minor local side effects, namely irritation, redness, and scaling of the treated area, in only two cases but did not observe systemic adverse effects (Mashiah et al., 2017).

Wang et al. (2017) treated proliferating IHs evaluating the effectiveness and the safety of 2% propranolol cream. In two patients the treatment response was graded scale 1, in 15 patients scale 2, in 17 patients scale 3, and in 6 patients scale 4. The Authors did not observe significant differences in location/size-related outcomes.

### Transdermal Propranolol

Several *in vitro* studies have been carried out to improve transdermal delivery of propranolol in order to reach deep IHs. Zeng et al. (2020) tested propranolol hydrochloride-loaded cubic nanoparticles (PRH-CNPs) on the abdominal skin obtained from Sprague Dawley rats and on endothelial cells of mouse hemangioendothelioma (EOMA). Smaller-sized PRH-CNPs demonstrated enhanced skin permeation towards EOMA cells when compared with the PRH solution.

Chopra et al. (2019) described the effects of a preparation composed by amorphous melts of propranolol incorporated into transdermal patches. The amorphous melts of propranolol were prepared using ionic liquids (ILs), which may be used as formulation additives, replacing oil or water, and may enhance transdermal penetration. In addition to a significant improvement in propranolol transdermal permeability from its amorphous melts, Chopra et al. (2019) observed also a reduction of skin irritation.

## Topical Timolol

### Topical Timolol Versus Oral Propranolol

Recently, timolol, a non-selective topical beta-blocker, was studies as a valid and safe option for the treatment of superficial, localized, small and uncomplicated IHs with less systemic absorption as well as absence of adverse effects (Khan et al., 2017).

According to Novoa et al. (2019), oral propranolol (1.0 mg/kg tablet once a day) and topical timolol maleate (0.5% eye drops twice a day) may equally produce a 50% or greater decrease in hemangioma diameter at 24 weeks (low-quality evidence). Although topical timolol was found as an effective treatment for superficial IHs, Khan et al. (2017) advised against its use when systemic treatment is justified by the anatomical location or size of the hemangioma that could lead to hepatic or cardiac impairment.

### Adverse Events

Eczema, ulcers, skin rashes, desquamation, and erythema are frequent adverse effects of topical timolol (Filoni et al., 2021). A large recent study in over 700 superficial IHs reported that 3.9% of patients treated with oral propranolol (2 mg/kg/day) presented systemic adverse events (AEs) while topical timolol treatment (0.5% hydrogel, three times daily) did not cause AEs (Wu et al., 2018).

### High-Risk Areas

Timolol is superior to watchful waiting in infants with superficial IH in high-risk areas. Cheng et al. (2020) studied infants of <1-year-old (within 13-month) which presented superficial IHs in high-risk areas. The IHs were smaller than 2 cm. Patients who received timolol showed significantly fewer complications than the control group, in which watchful waiting has been performed (4.2% versus 29%).

Lip represents a high-risk region for ulceration in IHs patients (Cheng and Friedlander, 2016). Xu et al. (2020) demonstrated that topical timolol administration was not effective for lip IHs compared to oral beta-blockers. Although very efficient and safe, therapy with oral propranolol is often not sufficient for IHs of lips and most cases needed additional therapy after systemic propranolol. A retrospective study showed that a pentagonal wedge resection of the segmental lip IHs is also an effective procedure for the treatment of lip IHs as well as for other vascular lip anomalies (de Castro et al., 2017). Other well-known surgical procedures for lip IHs include wedge resections and elliptical excisions (Zide et al., 1997) or rectangular block excision technique (Li W. Y. et al., 2011).

## Topical Carteolol

Carteolol is another nonselective beta-blocker, which shared with propranolol similar mechanisms of action; superficial and small IHs were successfully treated with topical carteolol. Xu et al. (2018) demonstrated a reduction of tumors and a response rate of 75% following treatment with carteolol. In addition, patients that showed complications, such as erythema and scarring, recovered without concern. This study suggested that topical carteolol is an effective, safe and noninvasive therapeutic alternative for IHs. Moreover, carteolol showed few complications. Head and neck hemangiomas, such as periorbital or cervical IHs, as well as localized and superficial hemangiomas, are particularly suitable for this therapy.

## Itraconazole

A single study by Chen et al. (2019) tested the effects of itraconazole on endothelial cells of mouse hemangioendothelioma (EOMA) cell line and infantile primary hemangioma endothelial cells (HemEC). Itraconazole blocked cellular proliferation in a dose-dependent manner and caused apoptosis in both cell lines. Moreover, itraconazole reduced vascular endothelial cell angiogenesis of HemEC and inhibited the expression of platelet-derived growth factor D (PDGF-D), thus reducing the PI3K/Akt/mTOR signaling, which is involved in the IHs pathogenesis. Gastrointestinal and liver disorders may appear as intraconazole side effects. Moreover, a possible interaction with any other concurrent medications should be considered (Chen et al., 2019).

## Sirolimus

Sirolimus is an inhibitor of mammalian target of rapamycin (mTOR), which is involved in the regulation of cell cycle and, therefore, of the vascular endothelial proliferation. Hutchins et al. (2017) observed a 3-year-old girl presenting diffuse cutaneous, hepatic and pulmonary IHs with progressive pulmonary hypertension which was treated with oral sirolimus (0.8 mg/m^2^ per dose twice daily). During the treatment, improvement of hepatic lesions and pulmonary hypertension was noted but the child died because of development of unexpected severe hypoglycemia. The authors, advocating the safety of sirolimus, however recommended its use in complicated cases with multi-organ involvement.

Le Sage et al. (2018) observed no improvement in patients with infantile hemangioma treated with sirolimus. The authors claimed that the absence of a lymphatic component in IHs may explain the low effectiveness of topical sirolimus in these lesions: this would explain its useful treatment for cutaneous manifestations of lymphatic malformations.

## Pulsed Dye Laser

Pulsed dye laser (PDL) is widely used for the treatment of IHs but its use is controversial. Ying et al. (2017) observed that both timolol maleate 0.5% cream and 595-nm pulsed dye laser (PDL) resulted in a significant clinical improvement of superficial IH during the proliferating phase.

The association of PDL with beta-blockers for the treatment of superficial and mixed IHs have appeared superior to PDL in efficacy and cost benefit (Asilian et al., 2015; Reddy et al., 2013). According to Valdebran et al. (2017), lasers are recommended for the treatment of superficial and thin IHs, whereas oral propranolol is mandatory for the therapy of deep IHs affecting the airways or obstructing the visual field. Finally, combined therapies may improve the outcome of mixed IHs and refractory superficial IHs (Valdebran et al., 2017).

## Infantile Hemangiomas With Involvement of Central Nervous System: PHACE and LUMBAR Syndrome

PHACE syndrome is characterized by a large (>5 cm) facial hemangioma which is associated with several congenital anomalies (Judd et al., 2007).

Instead, in LUMBAR syndrome a segmental IH in the lower body region is associated with urogenital, bony, anorectal, arterial anomalies and mielopathy (Golabi et al., 2014).

The risk of hidden arterial anomalies in LUMBAR syndrome requires a careful investigation before starting treatment with oral propranolol (Johnson and Smidt, 2014; Yu et al., 2017). In a recent European multicenter observational retrospective study, seven infants with large or segmental cutaneous IHs, involving the head, neck, lumbar or sacral area, were screened using MRI for PHACES or LUMBAR syndromes revealing intracranial or intraspinal IH. All patients underwent oral propranolol treatment for 6–14 months. All CNS lesions responded to treatment and five patients had a complete or almost complete resolution of the cutaneous IHs, thus demonstrating that propranolol can pass the blood-brain barrier. This study suggest that oral propranolol should be considered the first-line approach for intra-CNS IHs to avoid possible complications (Diociaiuti et al., 2020).

## Conclusion

The treatment of infantile hemangiomas is challenging even today. In our review, we described the last 5-year experiences with propranolol, the first line therapy for IHs treatment, and, in particular, with off-label drugs, such as topical β-blockers, including topical timolol and carteolol, steroids, itraconazole or sirolimus. Currently, oral propranolol is used in the majority of cases, but topical β-blockers can be preferred in superficial and uncomplicated forms. Oral and topical β-blockers have changed the prognosis of IHs, but some parents and physicians are reluctant to systemic therapies because of the risk of adverse events. Some alternative therapies are emerging, but data are not still enough to evaluate their efficacy and safety.

The need to lower as much as possible the risk of adverse events in pediatric population drives the search of other therapies than oral propranolol or steroids. We hope that further studies may confirm the safety of current therapies for IHs and expand the range of alternatives.
